# pH-controlled synthesis of sustainable lauric acid/SiO_2_ phase change material for scalable thermal energy storage

**DOI:** 10.1038/s41598-021-94571-0

**Published:** 2021-07-22

**Authors:** Shafiq Ishak, Soumen Mandal, Han-Seung Lee, Jitendra Kumar Singh

**Affiliations:** 1 Department of Architectural Engineering, Hanyang University, 1271 Sa 3-dong, Sangnok-gu, Ansan, 15588 South Korea; 2 Intelligent Construction Automation Center, Kyungpook National University, 80, Daehak-ro, Buk-gu, Daegu, 41566 South Korea; 3 Innovative Durable Building and Infrastructure Research Center, Department of Architectural Engineering, Hanyang University, 1271 Sa-3-dong, Sangnok-gu, Ansan, 15588 South Korea

**Keywords:** Chemistry, Energy science and technology, Engineering, Materials science

## Abstract

Lauric acid (LA) has been recommended as economic, eco-friendly, and commercially viable materials to be used as phase change materials (PCMs). Nevertheless, there is lack of optimized parameters to produce microencapsulated PCMs with good performance. In this study, different amounts of LA have been chosen as core materials while tetraethyl orthosilicate (TEOS) as the precursor solution to form silicon dioxide (SiO_2_) shell. The pH of precursor solution was kept at 2.5 for all composition of microencapsulated LA. The synthesized microencapsulated LA/SiO_2_ has been characterized by Fourier transform infrared spectroscopy (FT-IR), X-ray diffraction (XRD), X-Ray photoelectron spectroscopy (XPS), Scanning electron microscopy (SEM), and Transmission electron microscopy (TEM). The SEM and TEM confirm the microencapsulation of LA with SiO_2_. Thermogravimetric analysis (TGA) revealed better thermal stability of microencapsulated LA/SiO_2_ compared to pure LA. PCM with 50% LA i.e. LAPC-6 exhibited the highest encapsulation efficiency (96.50%) and encapsulation ratio (96.15%) through Differential scanning calorimetry (DSC) as well as good thermal reliability even after 30th cycle of heating and cooling process.

## Introduction

It is a great concern of the scientific community about the rising trend of the world’s energy consumption. Global energy demand is increasing rapidly, and fossil fuels consumption leads to higher greenhouse gas emission especially carbon dioxide (CO_2_), which will cause impacts on climate change, acid rain and human health^1^. International Energy Agency (IEA) report on World Energy Outlook 2019 estimates that the world energy demand will rise by 1.0% annually starting from 2018 to 2040^2^. Therefore, the research on energy storage has attracted significant attention for its effective use, storage and planning^3,4^. In recent years, thermal energy storage (TES) technology has aroused considerable concern about fuel energy shortages and environmental pollution^5^. Latent heat storage systems are considered as one of the most promising thermal energy technology, which relies on the absorption and release of energy of PCMs^6^. In other words, good thermal efficiency, and large storage capacity during the melting and solidifying process of PCMs will make them an efficient material in solar and industrial waste energy application^7–10^.

PCMs are commonly known as solid–solid and solid–liquid where they undergo phase transformation by turning their physical characteristics into solid or liquid^11^. Layered perovskites, dihydric phosphate salts, ammonium thiocyanate, polyatomic alcohol and polyurethane-based co-polymers are examples of the solid–solid PCMs^12^. Apart from that, the most common is solid–liquid PCMs which are classified into three different categories such as organic materials (paraffin and fatty acid), inorganic salts and metals/metallic alloys^13–15^. Organic PCMs i.e. paraffin and fatty acid have gained significant attention owing to their high storage capacity, excellent chemical stability and low cost^16,17^. Fatty acid-based PCMs can be used in TES applications attributed to their high latent heat, good thermal stability, chemical durability, non-corrosive, non-toxic and easy availability^12^.

However, fatty acids cannot be used directly as PCMs owing to leakage issues during phase transformation from solid to liquid i.e. melting which contaminate and pollute the external environment^18^. Moreover, fatty acid PCMs cannot be used directly as construction building materials (CBMs) owing to their high super-cooling and low thermal conductivity issues. During phase transformation (solid to liquid), the melted PCMs may react with the external environment of cement matrix in CBMs resulting in the initiation of corrosion on steel rebar and strength reduction, thereby serviceability of the building reduces^19^. Therefore, the microencapsulation techniques have been considered as a suitable solution to overcome the aforementioned issues. The microencapsulation techniques control the shifting rate of heat and decrease the reactivity of the PCMs with other materials^20^. There have been various encapsulation methods studied by researchers such as complex coacervation^21^, interfacial polycondensation^22,23^, in-situ polymerization^24,25^ and sol–gel process^26–29^. However, sol–gel is a suitable method for the microencapsulation of PCMs owing to its simple operation under low-temperature condition^30,31^.

Moreover, a significant volume of research works has been focused on the synthesis and characterizations of microencapsulated PCMs using various types of shell/encapsulation materials such as silicon dioxide^32,33^, polystyrene^34^, melamine–formaldehyde^35^, urea–formaldehyde^36^, polymethyl methacrylate^37^, polycarbonate^38^, styrene-methyl methacrylate copolymer^39^, and rigid polyurethane^40^. Fang et al.^41^ have synthesized flame retardant n-hexadecane with SiO_2_ shell through sol–gel method where they have maintained the pH of precursor solution at 2–3 by adding hydrochloric acid. In other studies, authors have synthesized the palmitic acid-based PCMs through sol–gel process where encapsulation was carried out by controlling the pH of precursor solution^42^. Li et al. have produced a stable paraffin/silicon dioxide/expanded graphite composite PCMs through sol–gel process maintaining a pH value of 2^43^. In an another study, Zhang et al.^23^ have synthesized PCMs via sol–gel process with n-octadecane as core material and SiO_2_ as the shell material keeping a pH at 2–2.5.

From the literature search, it is found that the pH of the precursor solution is an important parameter to produce microencapsulated PCMs. Therefore, in the present study different amounts of core material i.e., LA has been taken to synthesize the microencapsulated PCMs by maintaining the 2.5 pH of the precursor solution using hydrochloric acid. Besides, it is very important to understand the actual mechanism involving in the formation of microencapsulated PCMs through sol–gel method. Lauric acid (LA) has been chosen as the core materials whereas tetraethyl orthosilicate (TEOS) as the encapsulation material i.e., precursor solution. The characterization of the synthesized microencapsulated PCMs have been carried by Fourier transformed-infrared (FT-IR), X-ray diffraction (XRD), scanning electron microscopy (SEM), transmission electron microscopy (TEM), and X-ray photoelectron spectroscopy (XPS) while thermal properties by differential scanning calorimetry (DSC) and thermogravimetric analysis (TGA).

## Materials and methods

### Materials

Lauric acid (LA, C_12_H_24_O_2_), sodium lauryl sulphate (SLS, NaC_12_H_25_SO_4_), ethyl alcohol (EA, C_2_H_5_OH) and hydrochloric acid (HCl) were purchased from Daejung Chemical and Metals Co., Ltd., Gyeonggi-do, South Korea while Tetraethyl orthosilicate (TEOS, C_8_H_20_O_4_Si) from Acros Organics, Geel, Belgium. The melting point and purity of LA were 44.46 °C and 99%, respectively. Tetraethyl orthosilicate (TEOS, C_8_H_20_O_4_Si) acted as the precursor of encapsulating material for LA. The sodium lauryl sulphate (SLS, NaC_12_H_25_SO_4_) was used as an anionic surfactant. Hydrochloric acid (HCl) was used as the activator and ethyl alcohol (EA, C_2_H_5_OH) as the solvent.

### Procedure for microencapsulation of LA

The microencapsulation of LA was carried out in two steps. The first step was the preparation of precursor solution which includes hydrolysis and condensation of TEOS. The second step was the preparation of emulsion solution for the encapsulation process.

#### Preparation of precursor solution

The precursor solution was prepared by mixing 10 ml TEOS, 10 ml EA and 20 ml distilled water in a beaker as shown in Table 1. The pH of this solution was reduced from 6.5 to 2.5 using HCl. Once the pH of precursor solution was attained up to 2.5, the solution was stirred at 400 rpm and 50 °C for 30 min until a transparent solution of TEOS was obtained. This step is called hydrolysis of TEOS, which is amphiphilic in nature and favourable to encapsulate the LA^44^.

**Table 1:** Details composition for microencapsulation of LA.

Sample ID	Precursor solution				Emulsion solution		
	TEOS (ml)	Ethanol (ml)	Distilled water (ml)	pH	LA (g)	Distilled water (ml)	SLS (g)
LAPC-1	10	10	20	2.5	5	100	0.15
LAPC-2					10		0.20
LAPC-3					15		0.25
LAPC-4					20		0.30
LAPC-5					30		0.40
LAPC-6					50		0.60

#### Preparation of LA emulsion

Different amounts of LA i.e., 5, 10, 15, 20, 30, and 50 g were taken as PCMs as shown in Table 1. These amounts of LA were mixed with different amounts of SLS in 100 ml distilled water (Table 1) under vigorous agitation using a mechanical stirrer at 600 rpm and 65 °C for 2 h. The pH of the emulsion solution was measured and found to be 4–4.5.

#### Microencapsulation of LA

The microencapsulation of LA was carried out through sol–gel process. The hydrolysed TEOS precursor solution was added dropwise into the LA emulsion and stirred vigorously at 800 rpm and 65 °C for 6 h. The resultant solution i.e., emulsion + precursor was kept for cooling, and then, was filtered followed by ringing with alcohol to remove excess fatty acid. Finally, the residue was oven-dried at 40 °C for 24 h.

**Figure 1:**
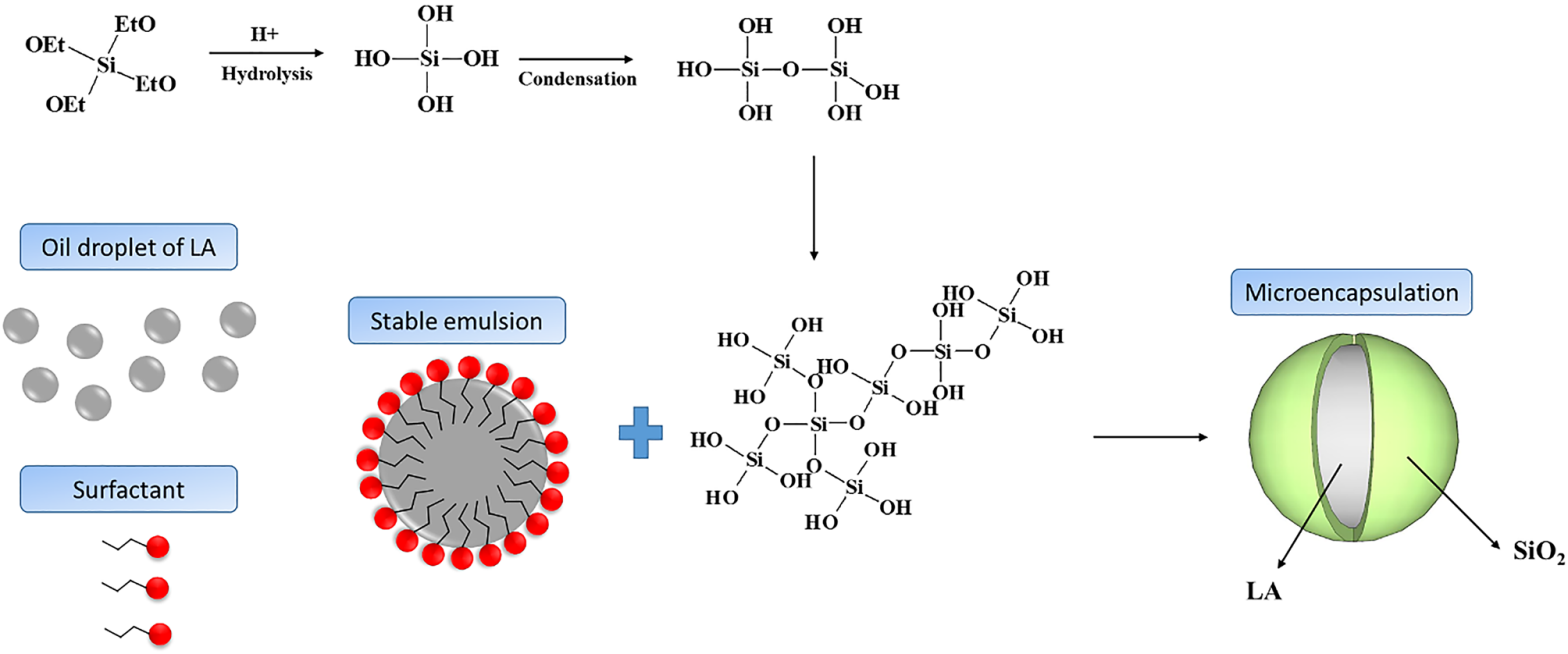
Schematic of the formation of microencapsulated LA with SiO_2_ shell.

The mechanism for the microencapsulation of LA is described in Fig. 1. The precursor solution i.e. TEOS would start to hydrolyse in the presence of acid solution and form orthosilicic acid (Si(OH)_4_). At the long term of stirring, further condensation reaction would occur. Alternatively, on another side, LA started to interact with SLS in an aqueous solution where SLS acted as an anionic surfactant carrying negatively charged ions. To form a stable emulsion solution, the hydrophilic head of the SLS molecules attach with the water molecules whereas the hydrophobic tails attach to the inner part of LA and separate away from water molecules. Thereafter the hydrolysed silica-sol solution was slowly added to the LA emulsion solution where the hydrophilic ends of hydrolysed silica slowly diffused and condensed onto the surface of LA. In the final step, polymerization reaction would take place resulting in microencapsulation of LA with SiO_2_.

### Characterization

FT-IR spectroscopy (Perkin Elmer UATR Two, United States of America) was used to analyse the chemical composition of pure LA as well as microencapsulated LA. The spectra were recorded ranging from 500 cm^−1^ to 4000 cm^−1^.

The XRD (D/MAX-2500, Rigaku, Japan) measurements were performed to analyse the phases present in the samples using 1.541 Å Cu-Kα radiation with 0.02° step width on continuous scanning mode. The XRD patterns were collected from 2θ = 5°–50° at 4°/min scan rate using 25 kV and 100 mA operating conditions.

The chemical states of the present element in the samples were analysed by XPS (Scienta Omicron R300, United States of America) with Al *K*α X-ray as the source of radiation. All the collected XPS spectra were calibrated with the adventitious carbon (C1s) peak (peak position: 284.6 eV). CASA XPS software was used to calibrate and deconvolute the scans as well as to correct the background (Shirley method) of the XPS peak. The best peak fittings were achieved by suitable Gaussian/Lorentzian fit.

The morphology and chemical compositions of the samples were investigated by SEM (TESCAN MIRA3, Czech Republic) coupled with an energy-dispersive X-ray (EDX) spectrometer at 15 kV as well as TEM (JEOL, JEM 2010, Tokyo, Japan).

Thermal properties (melting/solidifying temperature and latent heat) and thermal stability (thermal cycle) of the microencapsulated PCMs were investigated by DSC (TA Instrument, Discovery DSC, New Castle, United States of America) instrument from 20 °C and 60 °C with a 10 °C/min heating/cooling rate. The weight loss of the samples was investigated by a TGA analyser (TA Instrument, Discovery TGA, New Castle, United States of America) at 10 °C/min heating rate starting from 20 to 650 °C.

## Results and discussion

### FT-IR analysis

**Figure 2:**
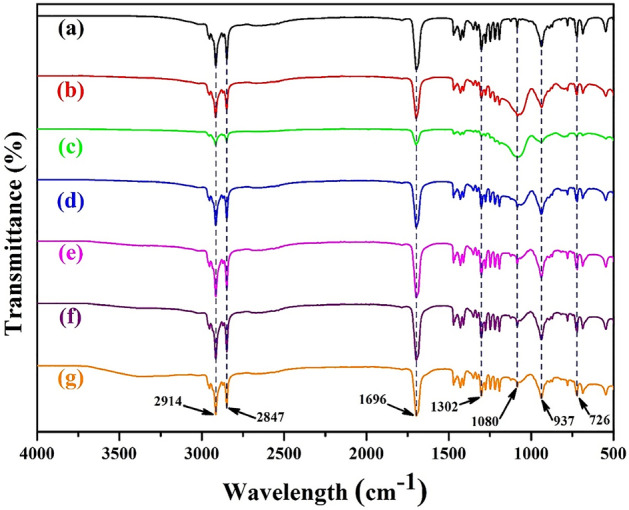
FT-IR spectra of (**a**) LA, (**b**) LAPC-1, (**c**) LAPC-2, (**d**) LAPC-3, (**e**) LAPC-4, (**f**) LAPC-5 and (**g**) LAPC-6.

The FT-IR analysis of bulk LA as well as microencapsulated LA/SiO_2_ are shown in Fig. 2. The bulk/pure LA has been chosen as reference materials. The FT-IR of pure LA shows four major peaks at 2914, 2847, 1696 and 937 cm^−1^ while two minor peaks at 1302 and 726 cm^−1^ as shown in Fig. 2a. The peaks at 2914 and 2847 cm^−1^ correspond to the stretching vibration of –CH_3_ and –CH_2_ group, respectively^45^. Alternatively, one intense peak at 1696 cm^−1^ exhibits the symmetrical C=O stretching vibration in LA^46^ while another peak at 937 cm^−1^ reveals the out of plane bending vibration for –OH group^45^. Moreover, two weak peaks appeared at 1302 cm^−1^ and 726 cm^−1^ are attributed to the stretching vibration of O–C group and plane bending vibration of C–H bond^47^. These peaks i.e., 2914, 2847, 1696, 1302, 937 and 726 cm^−1^ are also found in microencapsulated LA with SiO_2_ (Fig. 2b–g).

An interesting observation can be found in microencapsulated LA/SiO_2_ where one absorption peak at 1080 cm^−1^ is observed which corresponds to the asymmetric stretching vibration band of the Si–O–Si shell^23^. It depicts that the formation of SiO_2_ has occurred during the encapsulation of LA. Our finding is well corroborated with Zhang et al.^45^ works where the PCMs was encapsulated by a facile one-pot method with co-hydrolysis and co-condensation process of methyl-triethoxysilane (MTES).

The main absorption peak of SiO_2_ at 1080 cm^−1^ in lower core–shell ratio i.e., LAPC-1 (Fig. 2b) and LAPC-2 (Fig. 2c) is found to be highest in intensity and broadening revealing the thick and high amount of SiO_2_ compared to other samples. As the core–shell ratio is increased, the intensity of SiO_2_ absorption band is decreased confirming the thinning and lowering in amount of SiO_2_ formed onto the LA surfaces. Initially, the pH of the emulsion solution is found to be in between 4 to 4.5. However, once the precursor solution is mixed with the emulsion solution, the polymerisation would occur (Fig. 1) resulting in encapsulation of LA with SiO_2_ shell^48^. The slow condensation rate of SiO_2_ forms smooth and more compact layers onto the LA surface attributed to the sufficient time i.e., 6 h given for the reaction. Consequently, the low core–shell ratio might exhibit thicker shell formation compared to the higher core–shell ratio.

### XRD analysis

**Figure 3:**
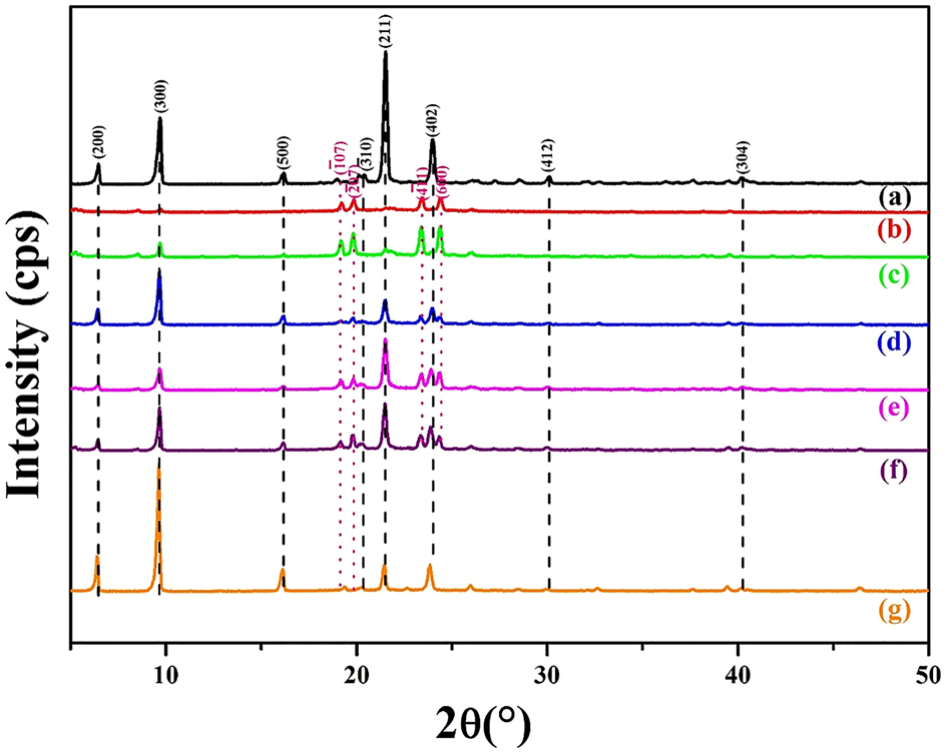
XRD patterns of (**a**) LA, (**b**) LAPC-1, (**c**) LAPC-2, (**d**) LAPC-3, (**e**) LAPC-4, (**f**) LAPC-5 and (**g**) LAPC-6.

The XRD results of pure LA powder and microencapsulated LA/SiO_2_ are shown in Fig. 3. The major XRD peaks of LA are found at 2θ = 6.461° (200), 9.699° (300), 16.199° (500), 20.381° ($\overline{3}$10), 21.52° (211), 23.98° (402), 30.121° (412) and 40.182° (304) which are well-matched with JCPDS No.: 038-1976 (C_2_H_24_O_2_, Lauric acid). These peaks are also found in the microencapsulated LA/SiO_2_ samples. Apart from these peaks, there are other peaks observed at 2θ = 19.22° ($\overline{1}$07), 19.86° ($\overline{2}$07), 23.421° ($\overline{4}$11) and 24.401° (600) correspond to LA (JCPDS No.: 008-0664). These peaks are well corroborated with Zhang et al. work where they have successfully synthesized microencapsulated n-octadecane with silica shell at pH 2–2.5^23^.

Broadening in XRD peaks of microencapsulated LA/SiO_2_ are observed attributed to the microencapsulation of core material i.e., LA with amorphous SiO_2_ shell around 2θ = 20°–30° as shown in supplementary Fig. S1. It can be seen from Fig. S1 that as the core material amount is increased (Fig. S1b–g), amorphousity is decreased attributed either to the thinning of shell wall or decrease in the amount of SiO_2_ onto the microencapsulated LA/SiO_2_ surfaces. Moreover, there is an interesting observation found in XRD peaks at (200), (300) and (500) where the intensity ratio of these peaks are increased as the core material is increased revealing the high amount of core materials in microencapsulated LA/SiO_2_.

It is observed by Wu et al. that the condensation rate is at its lowest point when the pH of the silica precursor is below 2.0 (isoelectric point) attributed to the neutral zeta (ζ) potential or zero surface charge^49^. At the neutral charge, the particles of the precursor solution tend to agglomerate, aggregate and flocculate. Thus, it is necessary to increase the pH of the precursor solution (pH > 2.0) to keep a stable solution with a good dispersion particle. In another study, pH at 2 is not recommended as the solution is prone to aggregate and flocculate^43^. For pH values above isoelectric point, the ζ potential values of silica become more negative^50^. Thus, it is suggested to keep the precursor solution pH greater than 2 where the charged particles are repelled to each other and avoid the agglomeration caused by the Van der Waals forces^48^ result in formation of uniform and compact layer of SiO_2_ on the surface of LA^49,51–53^.

### XPS analysis

**Figure 4:**
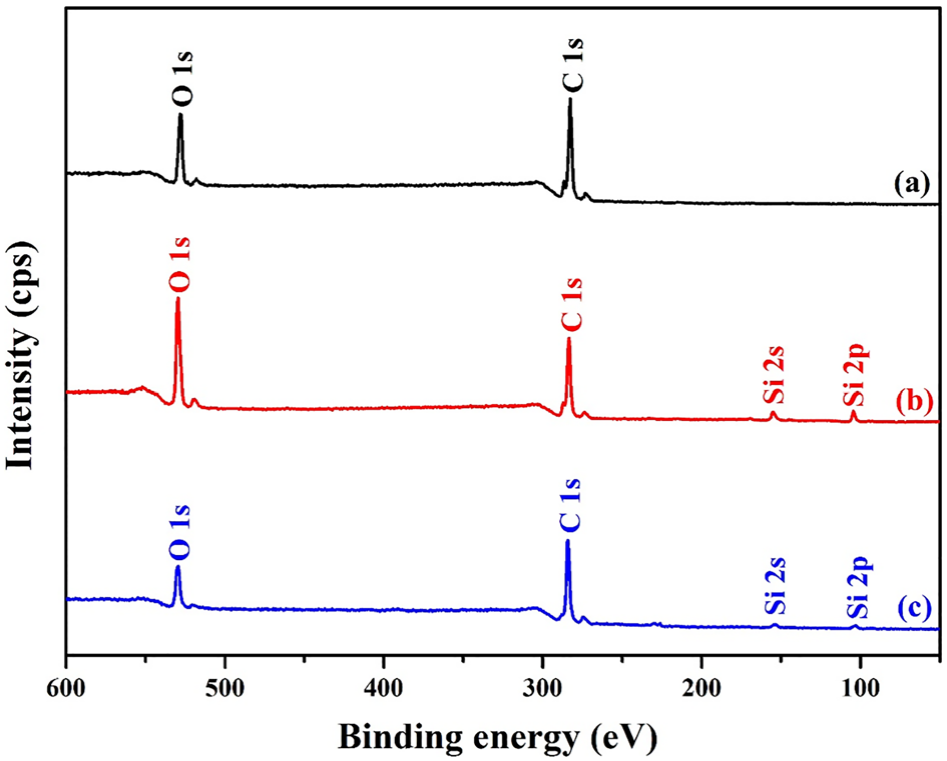
XPS survey scan of (**a**) LA, (**b**) LAPC-1 and (**c**) LAPC-6.

**Figure 5:**
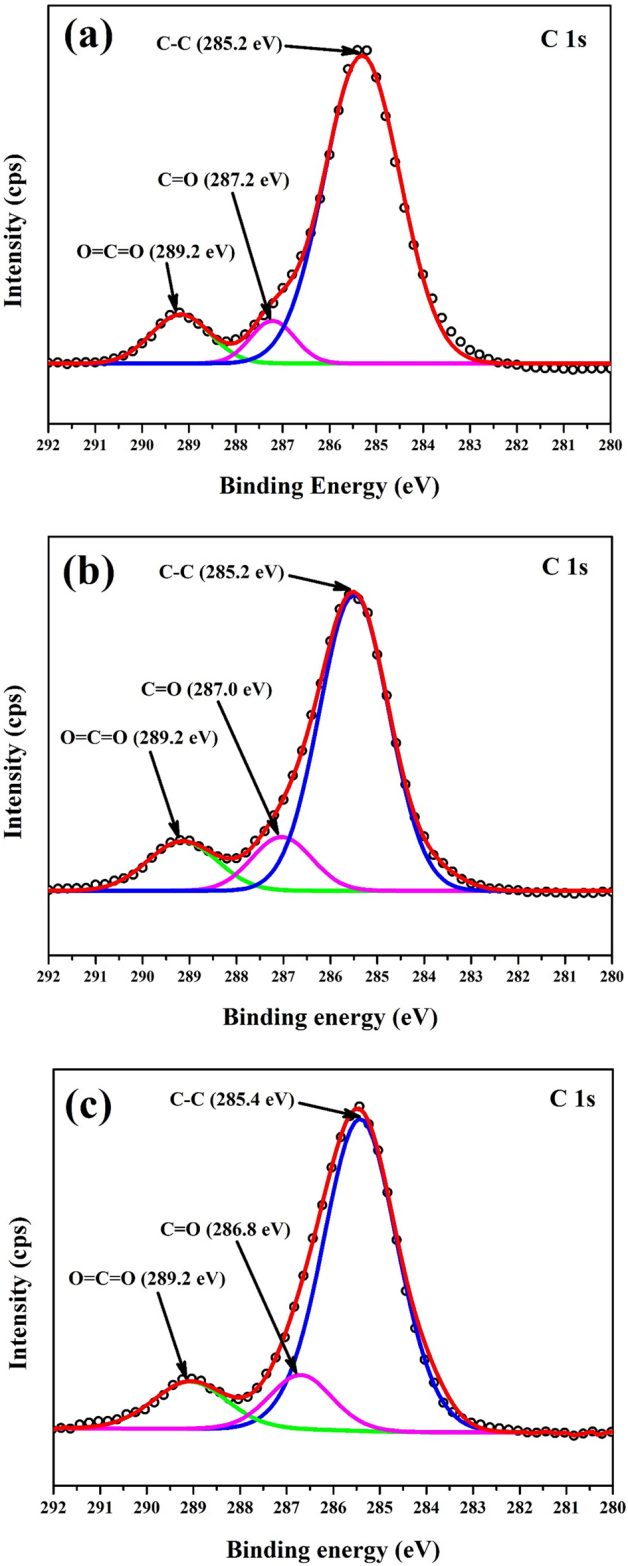
XPS spectra of C 1 s for (**a**) LA, (**b**) LAPC-1 and (**c**) LAPC-6.

**Figure 6:**
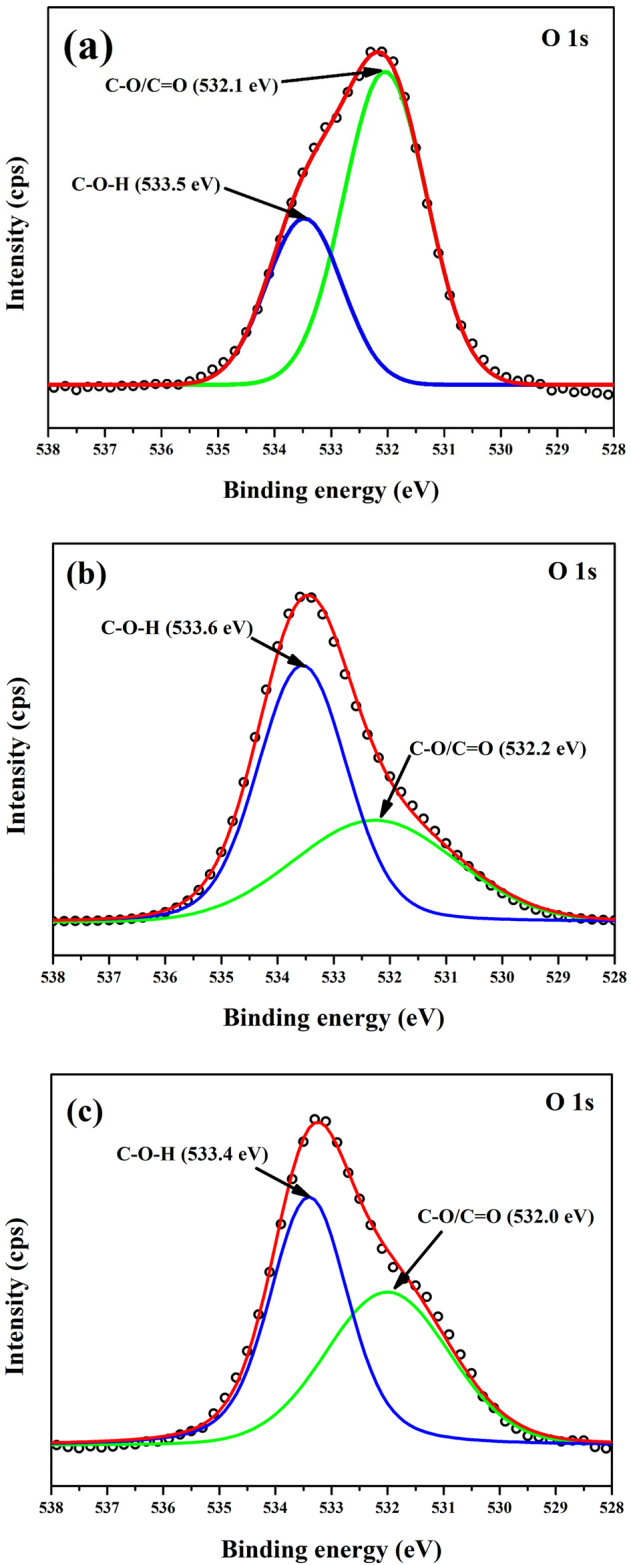
XPS spectra of O 1 s for (**a**) LA, (**b**) LAPC-1 and (**c**) LAPC-6.

**Figure 7:**
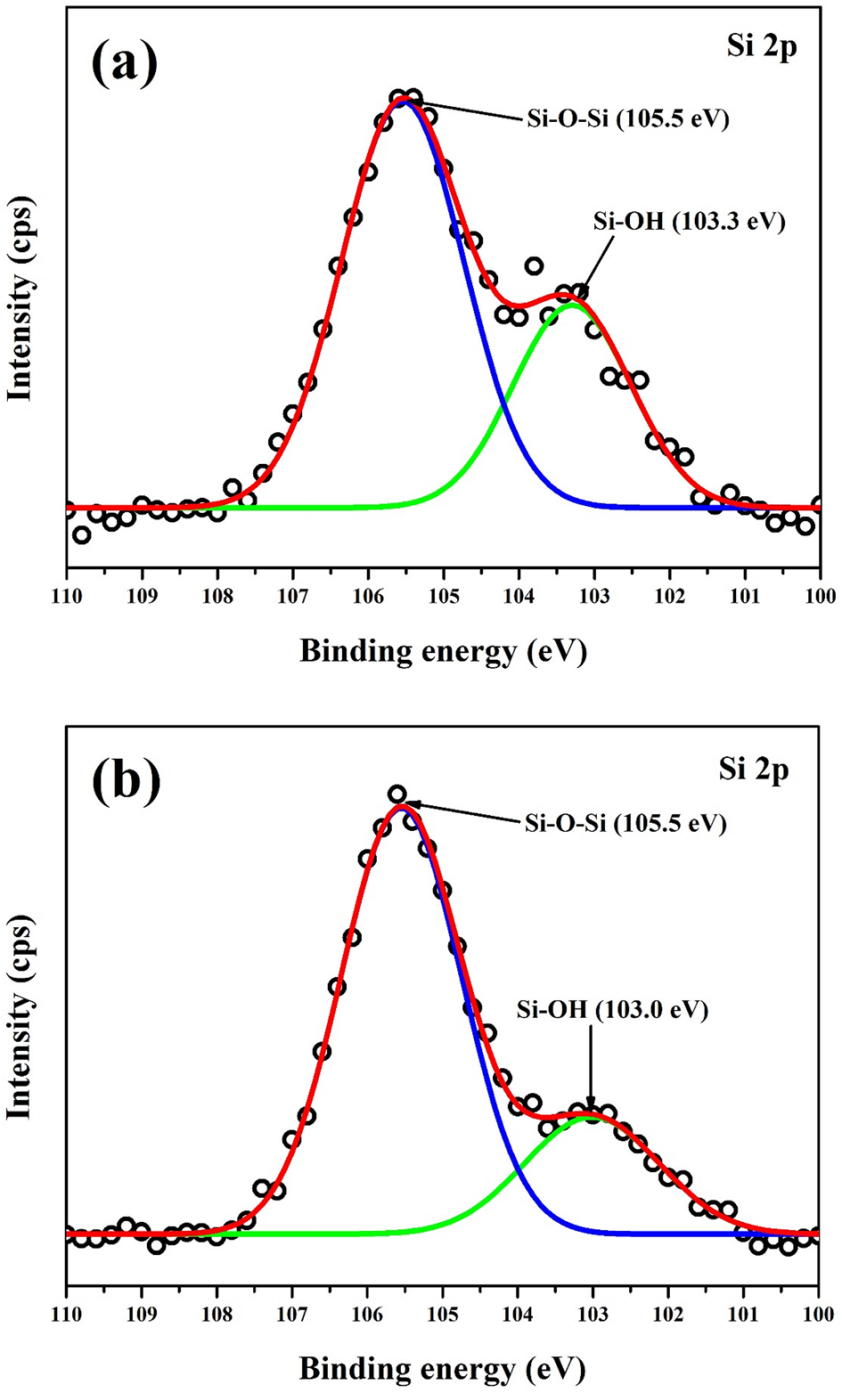
XPS spectra of Si 2p for (**a**) LAPC-1 and (**b**) LAPC-6.

The XPS survey scan of the LA, LAPC-1 and LAPC-6 samples are shown in Fig. 4. It reveals the presence of C 1 s and O 1 s in all samples as well as two other peaks of Si 2 s and Si 2p in LAPC-1 and LAPC-6 samples attributed to the microencapsulation of LA with SiO_2_ shell, which also corroborates with FT-IR and XRD results. The deconvolution of C 1 s, O 1 s and Si 2p are shown in Figs. 5, 6 and 7, respectively.

The XPS spectra of C 1 s (Fig. 5) are fitted with three different peaks which are C–C, C=O and O=C=O for all samples (LA, LAPC-1, and LAPC-6) which are attributed to the core material. The binding energy (BE) of C–C, C=O and O=C=O peaks in the present study are well corroborated with other researchers works^54–56^.

Figure 6 shows the XPS spectra of O 1 s with the presence of two peaks (Fig. 6a) of C–O–H (533.5 eV) and C–O/C=O (532.1 eV) for bulk LA whereas LAPC-1 (Fig. 6b) exhibits at 0.1 eV higher BE of C–O–H and C–O/C=O peaks attributed the adsorption of SiO_2_ onto the LA surface. There is broadening in C–O/C=O peaks observed for LAPC-1 (Fig. 6b) and LAPC-6 (Fig. 6c) attributed to the change in the chemical moiety owing to the presence of SiO_2_. The BE of these peaks are well fitted and satisfied with the earlier studies^54,57,58^.

Figure 7 shows the XPS spectra of Si 2p associated with the encapsulation of SiO_2_ onto the LA surface. No peak of Si 2p is found in bulk LA owing to the absence of SiO_2_. The Si 2p peak is fitted with Si–O–Si and Si–OH in microencapsulated LA/SiO_2_ samples. Both samples exhibited similar binding energy at 105.5 eV for Si–O–Si peak whereas Si–OH peak exhibits at 103.3 eV (Fig. 7a) and 103.0 eV (Fig. 7b) for LAPC-1 and LAPC-6, respectively. The intensity of Si–OH peak is higher in LAPC-1 compared to LAPC-6 owing to the formation of a thicker SiO_2_ wall.

### Morphology of the microencapsulated LA/SiO_2_

**Figure 8:**
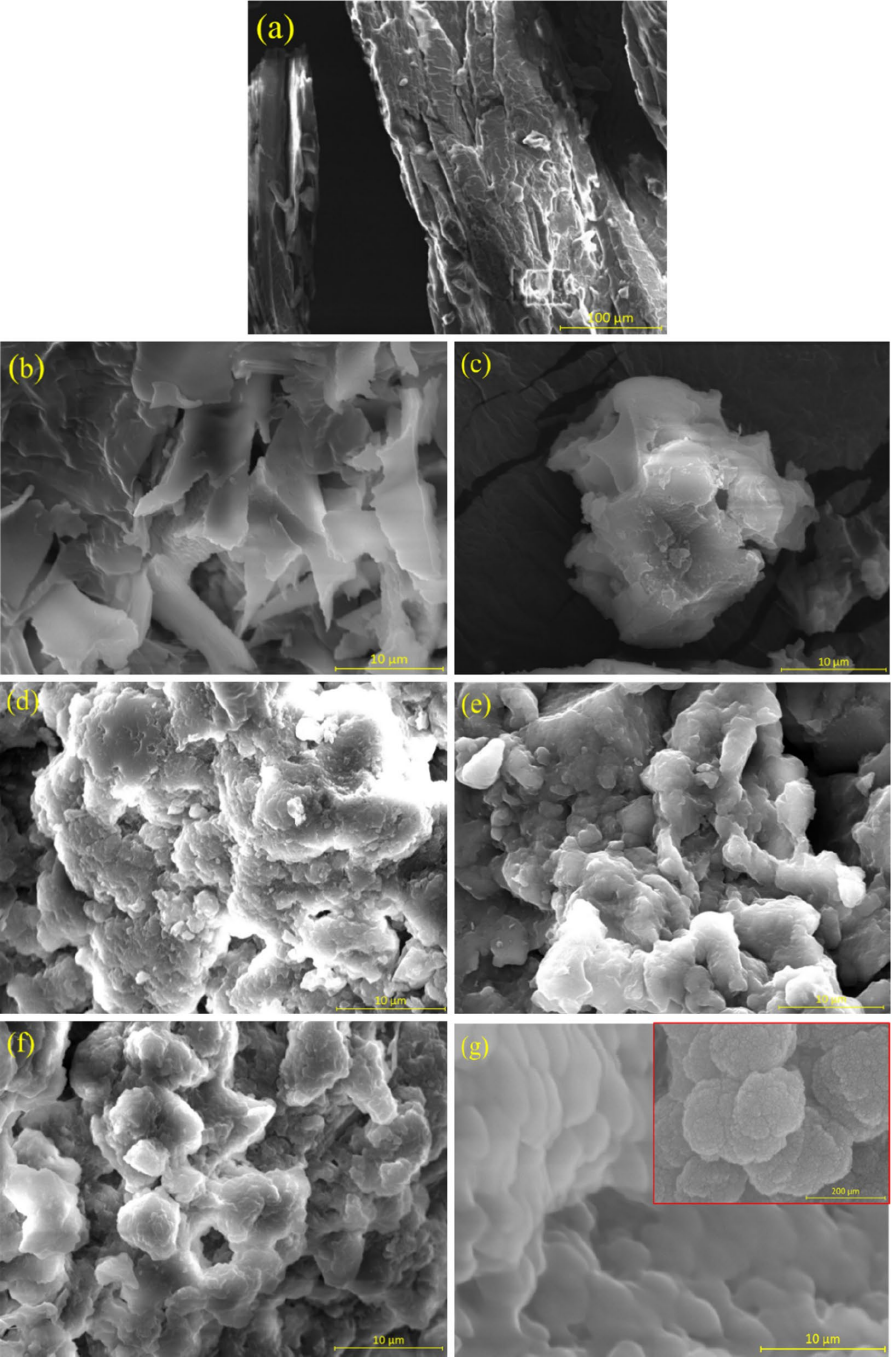
SEM of (**a**) LA, (**b**) LAPC-1, (**c**) LAPC-2, (**d**) LAPC-3, (**e**) LAPC-4, (**f**) LAPC-5 and (**g**) LAPC-6.

The morphology of LA and microencapsulated LA/SiO_2_ are shown in Fig. 8. It can be seen from Fig. 8a that LA exhibits irregular morphology with a rough surface. The particle of LA is around 200 µm in diameter and 500 µm in length. However, once the encapsulation has occurred, the morphology of the encapsulated PCMs has changed as shown in Fig. 8b–g.

**Figure 9:**
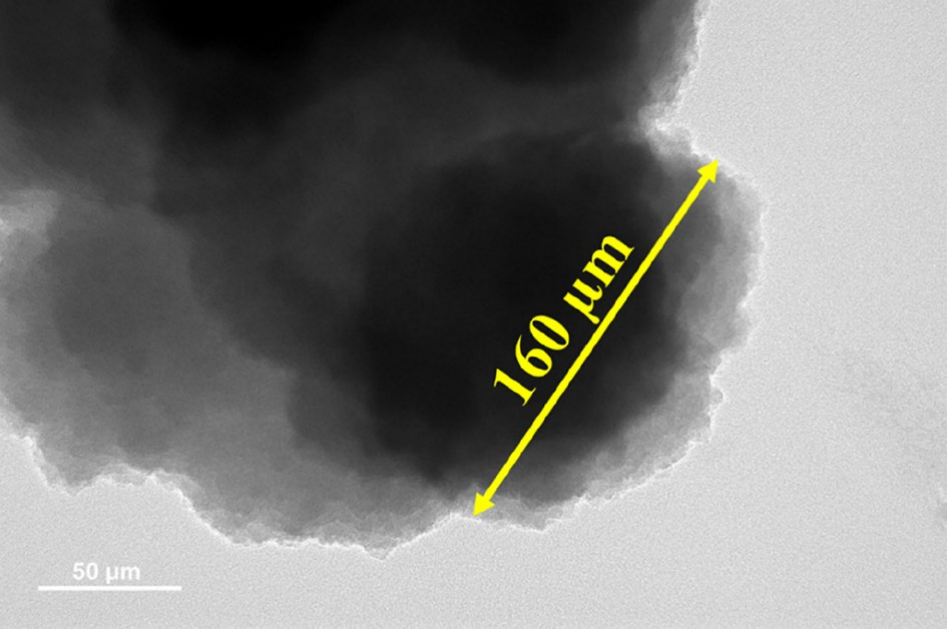
TEM of LAPC-6.

The morphology of LAPC-1 sample exhibited a dense structure of SiO_2_ (Fig. 8b) where the proper encapsulation cannot be identified^43^. It can be seen that at low amount of core material, the surface is more pronounced to form dense and agglomerated (Fig. 8b–d) structure^59^ owing to the significant amount of amorphous SiO_2_ which cover the LA surface^60^. The encapsulation of SiO_2_ on high amount of core materials i.e. LA leads to reduce the agglomeration^38^. The morphology of LAPC-4 (Fig. 8e) and LAPC-5 (Fig. 8f) samples are getting clear with the formation of globular particles even though the agglomerated structure still can be seen (Fig. 8e,f)^43,61,62^. Once the amount of core materials i.e. LA is reached up to 50% i.e., LAPC-6, the SEM image has shown more globular and well-defined structure^47^ (Fig. 8g inset). This result is well corroborated with TEM (Fig. 9) where light white colour SiO_2_ shell covered the core LA (black) uniformly. It can be seen from this Fig. that different globular microencapsulated LA/SiO_2_ particles are attached with each other (Fig. 8g). It can be explained that a high amount of LA reduces the number of Si–O–Si bonds by replacing Si–OH with Si–CH_3_ groups which decrease the connectivity of SiO_2_ network and make less porous structure^63^. Once the amount of LA increases i.e., high surface area, only a few silica oligomers will deposit on the LA surface and led to increase the particle size of microencapsulated LA/SiO_2_. It can be seen from Fig. 8g that the particle size of microencapsulated LA/SiO_2_ is greater than 150 µm which is well corroborated with particles size measured by TEM. The condensation rate of SiO_2_ is low in an acidic environment where it has significant time to encapsulate the LA^23^. At the low pH value, the self-assembly of SiO_2_ particles occurs owing to the condensation of silanol. Thus, smooth and compact morphology is observed which provides mechanical strength to the PCMs and prevent the leakage at higher temperature^64^. It is observed by Li et al. that if the pH of precursor solution is less than isoelectric point i.e., 2, then the surface of encapsulated PCMs become rough and porous^43^.

The EDS analysis of LA and microencapsulated LA/SiO_2_ are shown in Table 2. LA sample exhibited C and O attributed to the hydrocarbon. However, microencapsulated LA/SiO_2_ contain C and O as well as different content of Si. At low core material amount, Si is highest but once the amount is increased, Si content is decreased, and C is increased. This result suggests that at the low amount of core material, the formation of Si is thicker whereas, at high amount of core material, the thinning of SiO_2_ is occurred. Therefore, well-defined microencapsulation is observed in LAPC-6 sample (Figs. 8g, 9). There is no consistency in the amount of C and O due to the semi-quantitative analysis of EDS^65^. The presence of Si in the microencapsulated LA/SiO_2_ is well corroborated with the results of FT-IR, XRD and XPS where SiO_2_ is observed.

**Table 2:** EDS result of LA and microencapsulated LA.

Sample	Elements (Wt. %)		
	C	O	Si
LA	88.98	11.02	–
LAPC-1	27.77	30.58	41.65
LAPC-2	37.53	25.58	36.89
LAPC-3	41.36	28.30	30.34
LAPC-4	47.41	26.25	26.34
LAPC-5	58.19	17.27	24.54
LAPC-6	70.32	14.34	15.34

### Thermal performance of the microencapsulated LA with SiO_2_ shell

**Figure 10:**
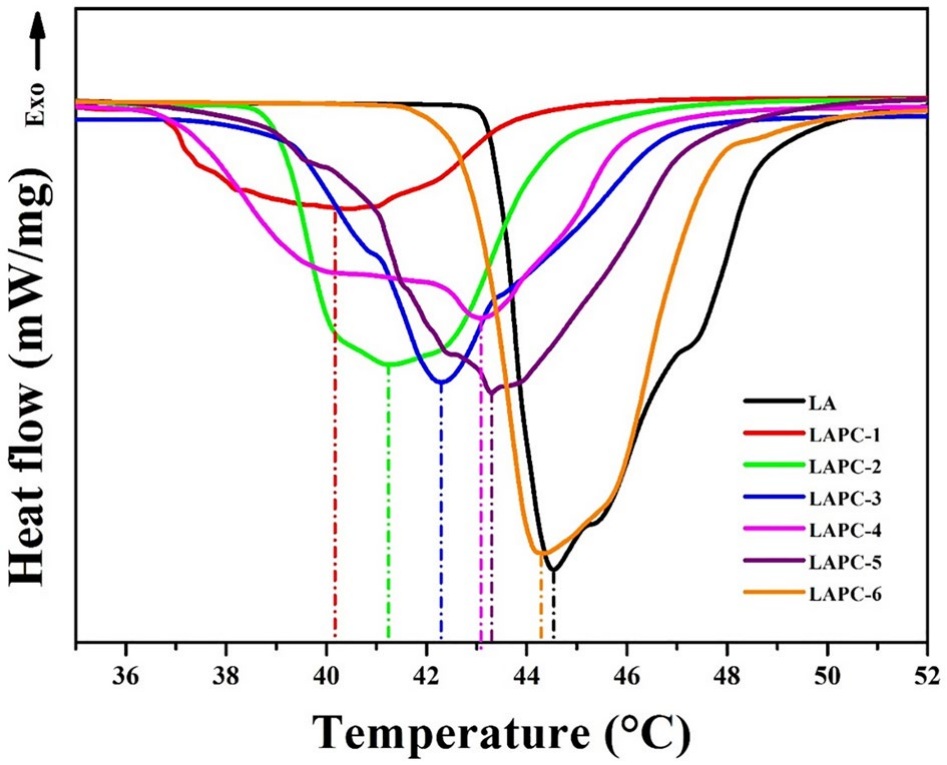
Melting curves of LA and microencapsulated LA/SiO_2_.

**Figure 11:**
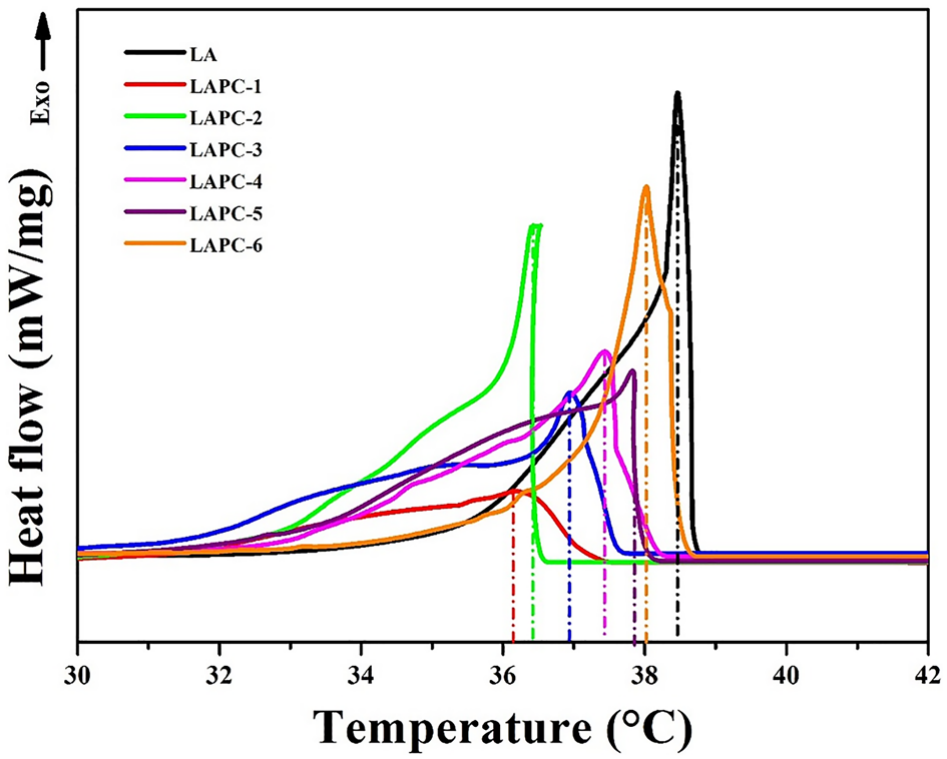
Solidifying curves of LA and microencapsulated LA/SiO_2_.

Melting and solidifying characteristics of microencapsulated LA/SiO_2_ are shown in Figs. 10 and 11, respectively, as well as the corresponding thermal data have been presented in Table 3. It can be seen from melting (Fig. 10) and solidifying (Fig. 11) curves that all samples exhibit different endothermic and exothermic peak temperatures. LA sample shows the highest melting i.e., 44.459 °C (Fig. 10; Table 3) and solidifying temperature i.e., 38.487 °C (Fig. 11; Table 3) as well as 166.74 J/g melting latent heat and 159.54 J/g solidifying latent heat (Table 3). This result suggests that the latent heat of LA is high, thus, it can be considered as prudent PCMs for the application in thermal storage^62^. When the amount of core material is less, the melting and solidifying temperatures, as well as the corresponding latent heat values are lower compared to LA attributed either to the lowest amount of LA where shell i.e., SiO_2_ act as an inert material or thickening of the shell wall. In this case, the movement of the LA molecules are limited and confined to a limited space by the shell^38,66–68^ or there is a possibility that microencapsulated LA/SiO_2_ have many empty shells without core materials^69^. The core material is responsible for the storage and release of thermal energy rather than shell materials. However, once the core material i.e., LA is increased, the melting and solidifying temperatures as well as the corresponding latent heat values are increased near to the value of bulk LA. LAPC-6 sample exhibited the highest melting temperature (44.295 °C) and latent heat of melting (160.91 J/g) as well as solidifying temperature (39.009 °C) and latent heat of solidification (152.82 J/g) among all microencapsulated LA/SiO_2_ where core material promotes the formation of thin SiO_2_ layer. This result is suggesting that microencapsulated LA/SiO_2_ can absorb/release the thermal energy during melting/solidifying process, thus, act as high latent heat storage material^59^. However, it can be seen that the melting and solidifying temperatures and latent heats of microencapsulated LA/SiO_2_ are lower than LA ascribing the weak interaction between the LA molecules and the inner surface wall of SiO_2_ shell^70^. The 6–7 °C lowering in melting and solidifying temperatures of LAPC-6 compared to LA (without encapsulation) suggesting that there is no strong interaction between LA and wall of the SiO_2_ shell, that results as the depression in phase change temperature of PCMs^71^.

**Table 3:** DSC of LA and microencapsulated LA/SiO_2_.

Sample ID	Melting		Solidifying		Encapsulation ratio (%)	Encapsulation efficiency (%)
	Temperature (°C)	Latent heat (J/g)	Temperature (°C)	Latent heat (J/g)		
LA	44.459	166.74	38.487	159.54	–	–
LAPC-1	40.176	64.203	36.138	57.110	38.51	37.18
LAPC-2	41.245	105.14	36.398	91.191	63.06	60.17
LAPC-3	42.299	113.44	36.984	101.73	68.03	65.95
LAPC-4	43.084	124.85	37.416	118.36	74.88	74.54
LAPC-5	43.291	155.80	37.843	144.86	93.44	92.15
LAPC-6	44.295	160.91	38.009	152.82	96.50	96.15

The microencapsulation ratio (R%) and efficiency (E%) of LA/SiO_2_ can be derived by following equations^23^:

1 $$ {\text{R}}\% = \frac{{\Delta {\text{H}}_{{\text{MEPCM, m}}} }}{{\Delta {\text{H}}_{{\text{PCM, m}}} }} \times 100 $$

2 $$ {\text{E}}\% = \frac{{\Delta H_{{\text{MEPCM, m}}} + \Delta H_{{\text{MEPCM, s}}} }}{{\Delta H_{{\text{PCM, m}}} + \Delta H_{{\text{PCM, s}}} }} \times 100 $$

where, ΔH_MEPCM,m_ and ΔH_PCM,m_ are the melting latent heat of the microencapsulated LA/SiO_2_ and LA while ΔH_MEPCM,s_ and ΔH_PCM,s_ is the solidifying latent heat of the microencapsulated LA/SiO_2_ and LA, respectively. Table 3 shows that LAPC-6 sample exhibited 96.50% encapsulation ratio and 96.15% encapsulation efficiency which are the highest among all microencapsulated LA/SiO_2_. The comparison in the encapsulation ratio and efficiency of the PCMs with the recent reported studies^72–75^ are presented in Table 4. It can be seen from this table that LAPC-6 has exhibited the highest encapsulation ratio and efficiency than earlier reported values.

**Table 4:** Comparison of the encapsulation ratio and efficiency with previous studies.

Core material	Shell material	Encapsulation ratio (%)	Encapsulation efficiency (%)	Reference
Lauric acid	Urea formaldehyde	26.26	28.03	^72^
Polytethylene gylcol	Silica	80.00	78.00	^73^
Paraffin	Titanium dioxide	81.37	81.77	^74^
Heptadecane	Calcium carbonate	49.8	49.7	^75^
Lauric acid	Silica	96.50	96.15	Our study

### Thermal stability and reliability of the microencapsulated LA/SiO_2_

**Figure 12:**
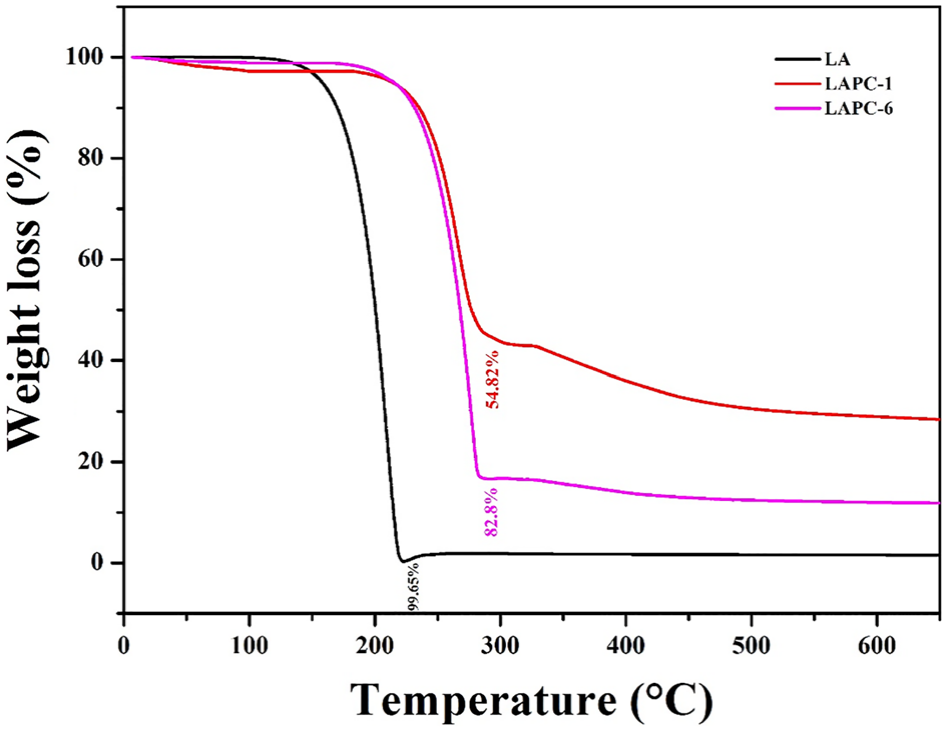
TGA curves of bulk LA and microencapsulated LA.

The thermal stability of PCMs is an important parameter for their application in real practices. The TGA of LA, LAPC-1 and LAPC-6 are shown in Fig. 12. LA sample shows a straight and smooth line up to 116 °C thereafter decomposition has started. Up to 99.65% weight loss has occurred at 220 °C for pure LA. However, LAPC-1 and LAPC-6 samples have started to decompose at 184 °C and maximum decomposition is found to be 54.82% and 82.80% around 288 °C, respectively. The decompositions of microencapsulated LA/SiO_2_ i.e., LAPC-1 and LAPC-6 have occurred via two steps. The first step from 184 to 288 °C is caused by mass loss of LA while the second step from 300 to 650 °C is attributed to the further condensation of silanol groups^62,67,76,77^. The decomposition of microencapsulated LA/SiO_2_ exhibit high decomposition/onset temperature and lower weight loss compared to LA. It suggests that SiO_2_ shell hinders or delays the decomposition and resulting in the high thermal stability of LA^69^. The weight loss of LAPC-6 sample is greater than LAPC-1 suggesting that the decomposition and weight loss mainly caused by the evaporation of LA^61^. Alternatively, in the case of LAPC-1, thick layer (Fig. 8b) and high content (Table 2) of SiO_2_ hinder the degradation process, thus, lower weight loss is observed. The carbonaceous-silicate charred layer was formed onto the LA surface which can protect the core materials and slow down the volatile products transferred during the thermal decomposition^64^.

**Figure 13:**
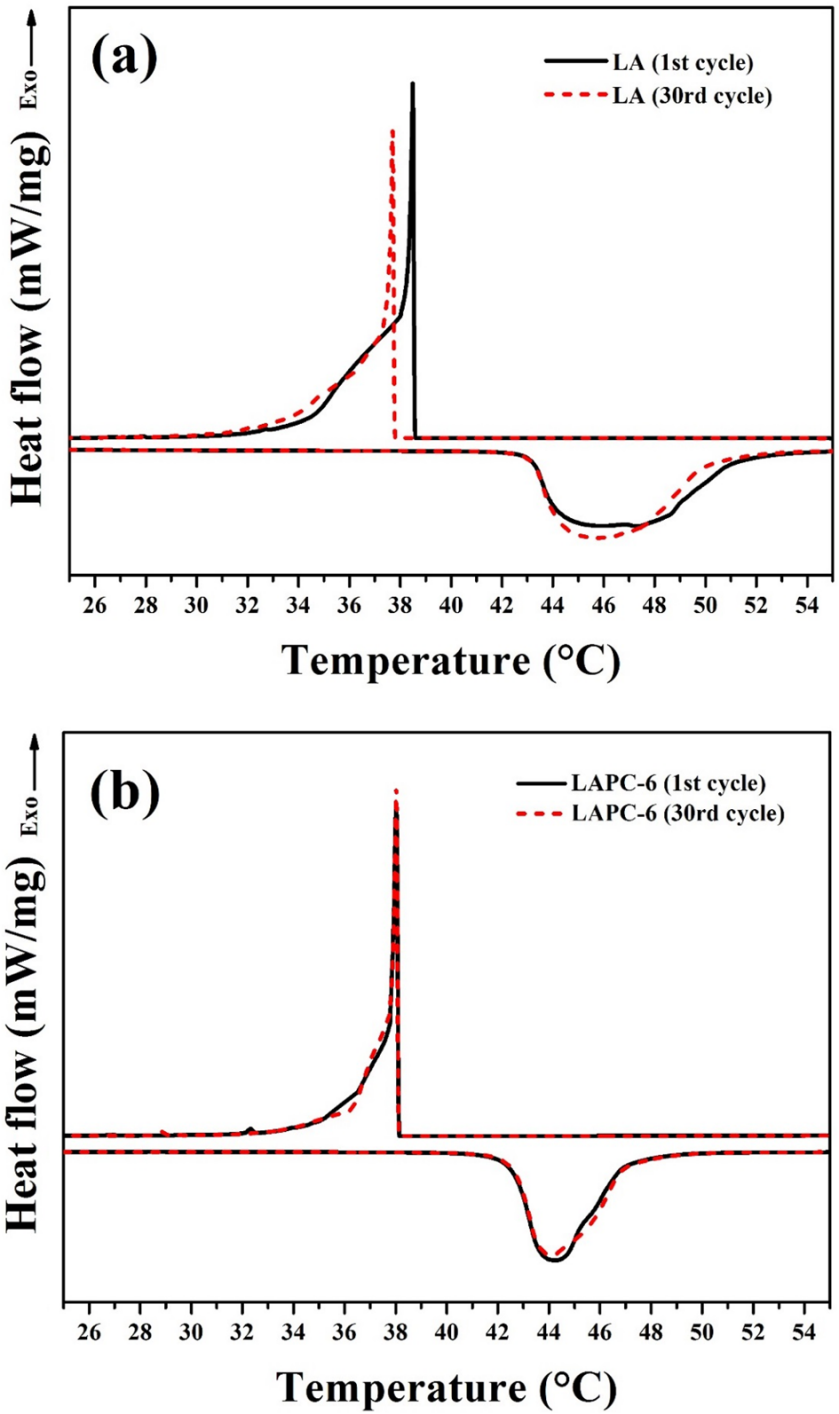
Thermal cycles of (**a**) LA and (**b**) LAPC-6 at 1st and 30th cycles of heating and cooling.

The other important parameter of the microencapsulated PCMs is the thermal reliability and consistency after being used for a certain period of time. There should be no significant difference in the thermal properties and stability after a certain duration that can affect the phase change temperature and latent heat capacity. Therefore, Fig. 13 shows the thermal reliability of LA and LAPC-6 after 30 cycles of heating and cooling process. The LA (without encapsulation) exhibits the melting and solidifying temperatures from 44.459 °C to 45.845 °C and 38.482 °C to 37.695 °C while latent heat changes from 166.74 J/g to 165.06 J/g and 159.54 J/g to 158.16 J/g from 1 to 30 cycles as shown in Fig. 13a, respectively. The melting and solidifying temperatures and latent heats of LA have decreased significantly. LAPC-6 sample shows the melting and solidifying temperatures change from 44.221 °C to 44.116 °C and 39.445 °C to 39.317 °C while the latent heat from 160.96 J/g to 160.75 J/g and 152.80 J/g to 152.71 J/g as shown in Fig. 13b, respectively. Negligible changes observed in melting and solidifying temperature and latent heat of LAPC-6 after 30 thermal cycles. Therefore, the microencapsulation of SiO_2_ is proven to be advantageous and it helps to sustain the thermal characteristic of the PCMs after a long period of application.

## Conclusions

In this study, LA has been chosen for microencapsulation with SiO_2_ shell. The microencapsulation of LA with SiO_2_ shell has been achieved successfully via sol–gel method using TEOS as precursor solution at 2.5 pH. Various kinds of analyses were conducted viz. FT-IR, XRD, XPS, SEM, TEM, DSC, and TGA to determine the properties of the microencapsulated LA/SiO_2_. FT-IR analysis has exhibited the presence of Si–O–Si peak at 1080 cm^−1^ for all microencapsulated LA while XRD analysis has shown the formation of hump at 2θ = 20°–30° associated with the amorphous SiO_2_ shell. XPS analysis of microencapsulated samples has shown the Si–O–Si and Si–OH peaks. SEM and TEM images of LAPC-6 has shown the formation of the fine-globular shape of microencapsulated LA/SiO_2_ whereas the samples with low amount of LA have exhibited the agglomeration. TEM analysis has shown that the particle size of LAPC-6 is found to be around 160 µm. LAPC-6 has exhibited the highest encapsulation efficiency and ratio among all microencapsulated LA/SiO_2_ with good thermal reliability.

## Supplementary Information

Supplementary Figure S1.
