# Supplementary material for: pH-controlled synthesis of sustainable lauric acid/SiO2 phase change material for scalable thermal energy storage
Source: Sci Rep. 2021 Jul 22;11:15012. doi: 10.1038/s41598-021-94571-0 (PMC8298577; doi:10.1038/s41598-021-94571-0)
Supplement: Supplementary file 1 — Supplementary Figure S1. [file 41598_2021_94571_MOESM1_ESM.docx]

**pH-controlled synthesis of sustainable Lauric acid/SiO_2_ phase change material for scalable thermal energy storage**

**Shafiq Ishak ^1^, Soumen Mandal ^2^, Han-Seung Lee ^1,^*, Jitendra Kumar Singh^3,^***

^1^ Department of Architectural Engineering, Hanyang University, 1271 Sa 3-dong, Sangnok-gu, Ansan 15588, Korea

^2^ Intelligent Construction Automation Center, Kyungpook National University, 80, Daehak-ro, Buk-gu, Daegu, 41566, Korea

^3^ Innovative Durable Building and Infrastructure Research Center, Department of Architectural Engineering, Hanyang University, 1271 Sa-3-dong, Sangnok-gu, Ansan 15588, Korea

**Fig. S1.** XRD patterns of **(a)** LA, **(b)** LAPC-1, **(c)** LAPC-2, **(d)** LAPC-3, **(e)** LAPC-4, **(f)** LAPC-5 and **(g)** LAPC-6 at 2θ=15°-35°.
